# Problems of Perinatal Mental Health Care in Tokyo, Japan

**DOI:** 10.14740/jocmr2371w

**Published:** 2015-10-23

**Authors:** Shunji Suzuki, Takashi Takeuchi, Tadaharu Okano, Naoki Kamiya, Takashi Sugiyama, Mayumi Ebine, Hideo Matsuda, Toshihito Suzuki, Takashi Okai, Satoru Takeda, Kazuhiko Ochiai, Katsuyuki Kinoshita

**Affiliations:** a Japanese Red Cross Katsushika Maternity Hospital, Tokyo, Japan; b Japan Association of Obstetricians and Gynecologists, Japan; c The Japanese Society of Perinatal Mental Health, Japan; d Japan Society of Obstetrics and Gynecology, Japan; e Tokyo Association of Obstetricians and Gynecologists, Tokyo, Japan

## To the Editor

Recently, a dramatic increase in pregnancies complicated by mental disorders has been observed in Tokyo, Japan [1, 2]. In 2014, the estimated number of deliveries complicated by mental disorders was 1,800 [2]. The rate of general hospital with psychiatric inpatient beds is only about 16% of the delivery facilities in Tokyo; however, about 36% of the deliveries with mental disorders were managed by these general hospitals. These rates are feared to lead to the tremendous burden of both obstetrics and psychiatric staffs of the general hospitals in Tokyo.

To know the reason why many deliveries with mild mental disorders are managed in a small number of general hospitals, we requested 85 private obstetric clinics to provide the reason why they introduced even deliveries with mild mental disorders to the general hospitals. Because many of the deliveries with mental disorders managed at the general hospital seemed to be not severe as necessary to be managed at the higher-order facilities. A total of 57 (67%) of them responded. The most common reason (26/57, 46%) was “We cannot examine the severity of mental disorders” and the second common reason was “It is difficult to take reservation of psychiatric clinics for pregnant women”. Therefore, they seemed to introduce all pregnant women suspected having mental disorders to the general hospitals. On the other hand, about 60% of the staffs of the psychiatric clinics in Tokyo seemed to be worried excessively about the influence of medications on both fetuses and pregnant women (Takeuchi and Okano, unpublished data). Therefore, some psychiatrists also seemed to introduce all pregnant women with mental disorders to the general hospitals.

For the proper management of perinatal psychosis, it is necessary to build a smooth cooperation system of obstetricians and psychiatrists. As the first step of the cooperation, the guidelines for the determination of severity of mental disorders by obstetricians those obtained a consensus between the obstetricians and psychiatrists are needed.
